# Public health round-up

**DOI:** 10.2471/BLT.25.010325

**Published:** 2025-03-01

**Violence in the Democratic Republic of the Congo**

Bulengo Camp on the outskirts of Goma in North Kivu, Democratic Republic of the Congo. The camp has become a refuge for over 120 000 internally displaced persons fleeing the conflict that recently intensified in the eastern part of the country.

**Figure:**
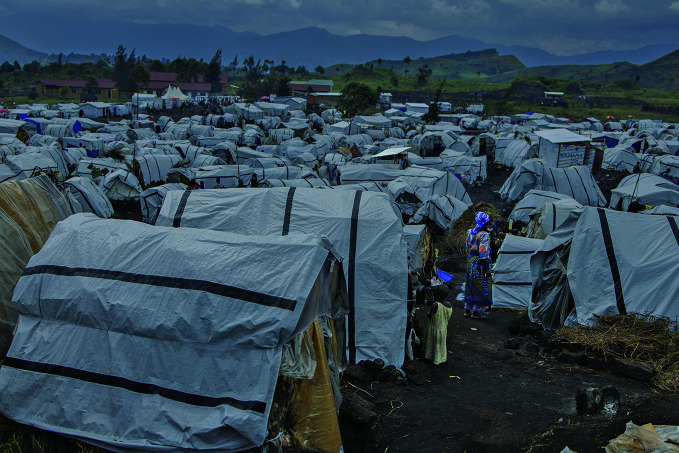
WHO/Guerchom Ndebo

## Withdrawal of the United States of America from WHO

The announced withdrawal of the United States of America (USA) from the World Health Organization (WHO) and its abrupt suspension of funding for key public health institutions is having a profound impact on global public health.

In a statement released on 21 January, WHO expressed regret at the decision, emphasizing the vital role played by the Organization in safeguarding global health and security, and expressed the hope that the USA would reconsider.

That hope was reiterated by Director-General Tedros Adhanom Ghebreyesus at the close of WHO’s Executive Board meeting on 11 February and again at a 12 February media briefing, where he noted that the USA’s withdrawal from other institutions is also causing concern.

The suspension of funding to the President's Emergency Plan for AIDS Relief (PEPFAR), for example, caused an immediate stop to human immunodeficiency virus (HIV) treatment, testing and prevention services in 50 countries. Although a waiver was subsequently granted for life-saving services, it did not include prevention services for some of the most at-risk groups.

“Despite the waiver, clinics are shuttered and health workers have been put on leave,” the Director-General said. WHO is gathering data on service disruptions and supporting countries with mitigation measures, including by filling gaps in supplies of antiretrovirals.

The Director-General also underlined the impact of the withdrawal on efforts to eradicate polio, respond to mpox epidemics, provide health care in Myanmar, and share vital information about the spread of avian influenza among dairy cattle in the USA.

“We ask the US to consider continuing its funding, at least until solutions can be found,” the Director-General said.

https://bit.ly/3CKel01

https://bit.ly/4hysXi1

## Sudan virus disease in Uganda

Uganda's Ministry of Health declared an outbreak of Sudan virus disease (SVD). The 30 January declaration followed confirmation from three national reference laboratories. The first person to be identified as infected developed symptoms between 20 and 21 January. As of 12 February, the government had reported nine confirmed cases, including one person who had died. More than 260 contacts had been identified and were being monitored.

According to a statement made by Director-General Tedros Adhanom Ghebreyesus on 12 February, WHO is providing surge support for surveillance, laboratories, logistics, infection prevention and control in hospitals, treatment centres and research.

National eemergency medical teams, trained by WHO, are also providing care for those infected. Additionally, WHO is providing critical supplies from the Organization’s logistics hubs in Nairobi and Dubai. To support the response, WHO released 2 million United States dollars (US$) from the WHO Contingency Fund for Emergencies, in addition to the US$ 1 million contributed by WHO to fund the initial response.

There are currently no authorized vaccines or therapeutics against the Sudan virus, but thanks to preparations made by the government, and a global research collaboration led by WHO, it was possible to start a trial of a candidate vaccine just four days after the outbreak was declared. A therapeutics trial will start as soon as national authorities provide approval.

https://bit.ly/3CKel01

https://bit.ly/41dQ0Jk

## Dire situation in the Democratic Republic of the Congo

Escalating violence in the eastern Democratic Republic of the Congo has given rise to mass casualties, population displacement and destruction of health infrastructure, worsening an already dire humanitarian crisis.

According to WHO, as of 12 February, more than 900 deaths had been reported, along with more than 4000 injuries. As of that same date only around one third of people who needed health services in North and South Kivu were able to receive them. These included pregnant women unable to reach health facilities for safe delivery. Meanwhile, the threat of infectious diseases including mpox and cholera was increasing.

Anticipating an escalation of violence, WHO had been working since November 2024 to position supplies including medicines and fuel. As of 12 February, those supplies were running out, and others were running dangerously low, a situation exacerbated by the USA decision to cut support and funding. WHO teams remained on the ground, but the security situation was limiting operations.

https://bit.ly/3CKel01

https://bit.ly/3WZR9BN

## Gaza ceasefire

The ceasefire in Gaza that began on 19 January and was still holding on 13 February made it possible for WHO and partners to provide some measure of humanitarian relief for the population.

As of 13 February, WHO had sent in 139 trucks with medical supplies for 1.6 million people and was positioning medical supplies at health facilities. Additionally, WHO had supported the evacuation of 414 patients.

According to WHO’s 12 February media briefing, the Organization had also sent emergency supplies to hospitals in response to the escalating violence in the West Bank.

The health challenges ahead are immense. The entire population of Gaza has faced multiple displacements. More than 46 600 people have been killed and over 110 000 have been injured. Only half of Gaza’s 36 hospitals remain partially operational, nearly all hospitals are damaged or partly destroyed, and just 38% of primary health-care centres are functional. An estimated 25% of those injured – around 30 000 people – have life-changing injuries and will need ongoing rehabilitation.

https://bit.ly/3CRlHyO

https://bit.ly/3CKel01

## Progress on neglected tropical diseases

Two countries announced significant progress towards eliminating neglected tropical diseases.

Niger met the criteria for onchocerciasis elimination, making it the fifth country globally and the first country in the African Region to be acknowledged by WHO for interrupting transmission of the parasite *Onchocerca volvulus*.

A parasitic disease, onchocerciasis (commonly known as river blindness) is the second leading infectious cause of blindness worldwide, after trachoma. It is transmitted to humans through the bites of infective black flies.

Guinea also took a significant step forward, by eliminating the gambiense form of human African trypanosomiasis as a public health problem. Human African trypanosomiasis (HAT), or sleeping sickness, is a vector-borne parasitic disease caused by infected tsetse flies. Symptoms include fever, headaches, joint pain and, in advanced stages, neurological symptoms like confusion, disrupted sleep patterns and behavioural changes. Without treatment, HAT is usually fatal.

This form of human African trypanosomiasis, the only type transmitted in Guinea, is the first neglected tropical disease to be eliminated in the country.

https://bit.ly/3WYfuIj

https://bit.ly/4hVCSOr

## Emergency health appeal

WHO called for US$ 1.5 billion for its 2025 Health Emergency Appeal to support life-saving health interventions worldwide.

The appeal was launched on 16 January by the Director-General in response to an unparalleled global health crisis in which 305 million people are in urgent need of humanitarian assistance in 2025.

The appeal outlines the critical priorities and resources needed to address 42 ongoing health emergencies, including 17 Grade 3 crises – the most severe emergencies requiring the highest level of response.

https://bit.ly/4hQAA32

## Paediatric cancer initiative

WHO and St. Jude Children’s Research Hospital began distribution of otherwise inaccessible childhood cancer medicines through the Global Platform for Access to Childhood Cancer Medicines.

According to an 11 February media release, shipments of cancer medicines began with Mongolia and Uzbekistan as part of a six-country pilot phase. Shipments to Ecuador, Jordan, Nepal, and Zambia are to follow, the aim being to treat 5 000 children across 30 hospitals by the end of the year. The six countries in the pilot phase will receive an uninterrupted supply of quality-assured childhood cancer medicines at no cost.

The overall goal of the initiative is to reach 50 nations in the next 5 to 7 years, eventually providing medicines for the treatment of approximately 120 000 children with cancer in low- and middle-income countries (LMICs). It is estimated that 400 000 children develop cancer every year. Close to 90% of them live in LMICs, where survival rates are less than 30%, compared with over 80% in high-income countries.

St. Jude has committed to a six-year investment to launch the Global Platform in partnership with WHO, the United Nations Children’s Fund and the Pan American Health Organization Strategic Fund.

https://bit.ly/4hAo0Fo

**Cover photo**

A mother carrying her baby awaits medical assistance in a WHO tent in Mecufi, Mozambique after both her home and the local hospital were destroyed by Cyclone Chido.

**Figure:**
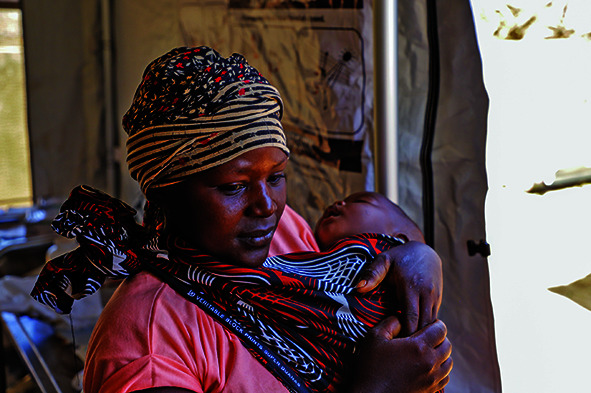
(WHO/Tiago Zenero)

**Looking ahead**

3 March. World Hearing Day 2025. https://bit.ly/40ZErUC

25–27 March. Second Global Conference on Air Pollution and Health. Cartagena, Colombia. https://bit.ly/4jUySjc

6–8 May. Particle Pathways Conference: exploring the nexus of indoor air pollution and airborne diseases. Gaeta Castle, Gaeta, Italy. https://bit.ly/41bqKDs

